# Are the Biological and Biomechanical Properties of Meniscal Scaffolds Reflected in Clinical Practice? A Systematic Review of the Literature

**DOI:** 10.3390/ijms20030632

**Published:** 2019-02-01

**Authors:** Chanuka D. S. Ranmuthu, Charindu K. I. Ranmuthu, Jodie C. Russell, Disha Singhania, Wasim S. Khan

**Affiliations:** 1 School of Clinical Medicine, Addenbrooke’s Hospital, University Of Cambridge, Cambridge CB2 0SP, UK; cdsr2@cam.ac.uk (C.D.S.R.), ckir2@cam.ac.uk (C.K.I.R.), jcr64@cam.ac.uk (J.C.R.), ds781@cam.ac.uk (D.S.); 2 Division of Trauma & Orthopaedic Surgery, Addenbrooke’s Hospital, University of Cambridge, Cambridge CB2 0QQ, UK

**Keywords:** meniscus, scaffold, knee, humans

## Abstract

The aim of this PRISMA review was to assess whether the CMI and Actifit scaffolds, when used in clinical practice, improve clinical outcomes and demonstrate the ideal biological and biomechanical properties of scaffolds: being chondroprotective, porous, resorbable, able to mature and promote regeneration of tissue. This was done by only including studies that assessed clinical outcome and used a scale to assess both integrity of the scaffold and its effects on articular cartilage via MRI. A search was performed on PubMed, EMBASE, Scopus and clinicaltrials.gov. 2457 articles were screened, from which eight studies were selected: four used Actifit, three used CMI and one compared the two. All studies reported significant improvement in at least one clinical outcome compared to baseline. Some studies suggested that the scaffolds appeared to show porosity, mature, resorb and/or have possible chondroprotective effects, as assessed by MRI. The evidence for clinical translation is limited by differences in study methodology and small sample sizes, but is promising in terms of improving clinical outcomes in the short to mid-term. Higher level evidence, with MRI and histological evaluation of the scaffold and articular cartilage, is now needed to further determine whether these scaffolds exhibit these useful properties.

## 1. Introduction

The menisci are two crescent-shaped discs of fibrous cartilage, consisting of 72% water, 22% collagen that is predominantly type I and 0.8% glycosaminoglycans (primarily chondroitin-6-sulfate) [1]. The components of this extracellular matrix are synthesised and maintained by fibroblast-like cells around the periphery and fibrochondrocytes more centrally. They perform numerous functions and the interactions between its constituents allow it to withstand compressive loads [2]. These constituents also have important biomechanical properties that allow them to withstand the stresses that they encounter in the knee during the six degrees of freedom, including adopting the shape of the femoral condyles and tibial plateau, increasing contact area in the knee joint and occupying this area, protecting articular cartilage [3]. They are necessary for load transmission and enhancing the stability of the knee, particularly when it is anterior cruciate ligament-deficient [4]. As vascularisation is restricted to the peripheral meniscus, healing can only occur in this region, whilst other areas do not have the blood supply to recover [5]. Hence, meniscal tears cause severe pain and discomfort and often require surgical intervention.

Meniscal tissue was previously believed to have no function and therefore injury was often treated by meniscectomy. However, loss of meniscal tissue has been shown to increase peak local contact stresses [6,7] and, due to its protective role for tibial cartilage, cause a dramatic increase in the risk of joint degeneration and the need for arthroplasty in the future [6]. Despite partial meniscectomy having more favourable outcomes as less tissue is removed [8], there is still a risk of the complications seen in meniscectomy and neither of the treatments encourage the regeneration of the meniscal tissue. There is a body of research looking into meniscal allograft transplantation (MAT) as a solution to complications following meniscectomy, with positive results at follow up. However, MAT aims to restore the entire or near entire meniscus so any remaining parts of the original meniscus must be removed before insertion [9].

Meniscal scaffolds can be used in patients who have had a partial meniscectomy, where a peripheral rim exists for attachment [10]. Meniscal tissue engineering is based around forming a matrix that is a permissive environment for tissue regeneration. There are two commercially available scaffolds – one biologic and one synthetic polymer. The Collagen Meniscus Implant (CMI, Ivy Sports Medicine GmbH, Gräfelfing, Germany) is made from type I collagen of bovine Achilles tendon, whose fibres are purified and swollen in the presence of equal parts hyaluronic acid (HA) and chondroitin sulfate. HA has been shown to suppress nitric oxide (NO) production in rabbits and, considering that NO donors cause meniscus degeneration, it follows that HA helps in meniscal healing [11,12]. Stone et al. (1992) demonstrated evidence of infiltration of the scaffold by fibrochondrocytes in 24 mixed breed dogs [13]. Collagen implants are resorbed both by active cellular resorption via multinucleated giant cells, particularly in the first weeks, and alternative pathways that are more evident later on, as seen in canine studies [14]. 

The synthetic polymer scaffold (Actifit, Orteq Bioengineering, London, UK) is composed of 80% soft polyester segments for flexibility and 20% stiff polyurethane segments for mechanical strength. The polyester segments are biodegradable and found in many existing medical products (e.g., sutures), with hydrolysis expected to take up to 6 years [15]. The polyurethane segments are semi-degradable and more stable but are much smaller than cells, hence removed by macrophage phagocytosis, as shown in rabbits. The study demonstrated that after three years, the polyurethane foam appeared to have resorbed completely [16]. Tissue ingrowth is then said to arise from blood vessel infiltration of the honeycomb structure of the Actifit scaffold [17]. Tissue growth has been demonstrated in vivo as well, for example, Tienen et al. (2006) displayed that the implant had been infiltrated with fibrovascular tissue and at 6M contained tissue resembling cartilage containing both proteoglycans and collagen type II in the matrix [18]. Aromatic polyurethanes have received negative attention as they may have toxic degradation products [15]. Thus, Actifit uses an aliphatic polyurethane that releases putrescine instead, which is found in the body normally. As a result of the ratio of constituents, the biomechanical properties should be adequate to provide protection of the articular cartilage as the menisci would [3].

Ideally, a scaffold would have a number of properties. For one, it should be porous to allow the infiltration of growth factors throughout the scaffold [18]. It should also create mechanical stimuli, which would encourage tissue to regenerate [19]. Further, the scaffold’s ability to degrade and resorb is important, as it allows the scaffold to eventually disappear once tissue repair has been completed. It is also desirable for the scaffold to be able to mature over time. This is thought to improve mechanical properties [20]. These mechanical properties should provide protection of the articular cartilage [3].

Previous reviews have evaluated and described these scaffolds in preclinical and clinical settings [3,15]. Our aim was to evaluate whether the biological and biomechanical properties that CMI and Actifit scaffolds are designed to have are evident in clinical practice by assessing clinical and radiological outcomes. The precise biochemical composition of these scaffolds allows for such properties and if they are not evident once the scaffold is implanted, this could represent a potential area of improvement to further optimise clinical outcomes. The scaffold also has effects on articular cartilage, which studies often fail to evaluate sufficiently. For this reason, we included studies which use both a scale to measure scaffold integrity on MRI and a scale to evaluate the articular cartilage. This would provide a more complete assessment of the outcomes of these scaffolds than what is already present in the literature. We followed the preferred reporting items for systematic reviews and meta-analysis (PRISMA) guidance [21].

## 2. Results

**Figure 1:**
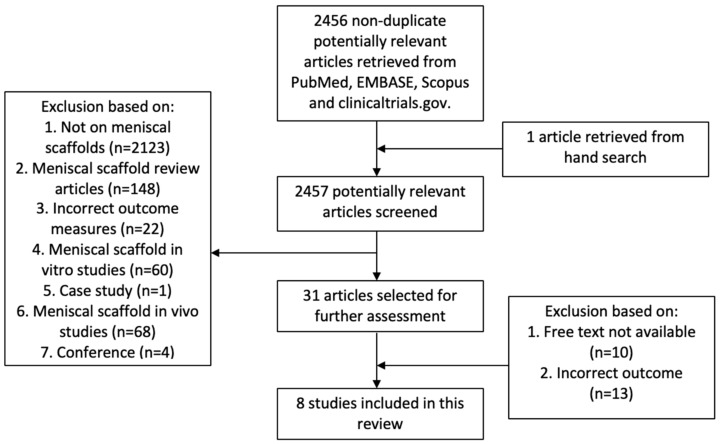
The study selection flow diagram.

Papers were excluded for a range of reasons, including not meeting the inclusion criteria, and these are listed in Figure 1. Included studies must have clearly reported clinical outcome scores along with MRI assessment using the Genovese score, as well as either a Yulish score or an ICRS score. The papers that did not meet these criteria are referred to as ‘incorrect outcome’ in the flow diagram. The literature search and screening process, shown in Figure 1, resulted in eight studies being selected for inclusion in this systematic review. As a result, this review includes three papers investigating CMI scaffolds [22,23,24], four investigating Actifit scaffolds [25,26,27,28], and one paper looking at both [29]. This included one paper which was found during the hand search and met the inclusion criteria [25]. One paper, Zaffagnini et al. (2011), compared CMI scaffold treatment to partial medial meniscectomy [23]. For this paper, the CMI scaffold group was only used for this review. Two studies were therefore prospective comparative studies [23,29]. All other studies were case series. There was a lack of randomised controlled trials found in the literature. Data from these eight studies are summarised in Table 1, Table 2, Table 3 and Table 4. Table 1 reports the details of each study, Table 2 summarises the clinical outcomes of each study, Table 3 shows MRI results and Table 4 shows the second-look arthroscopy and histology results of each study along with any complications/failure rates.

### 2.1. Coleman Methodology Score

The Coleman Methodology Score was used to assess the quality of the studies included [30]. The mean CMS and standard deviation was 61.125 ± 6.73, and the range was 49–68. The maximum score achievable in the CMS is 100. The mean CMS and standard deviation for Part A was 34 ± 6.28 and for Part B was 27.125 ± 2.59. A full breakdown of the scores achieved by each study can be found in Table S1 in the ‘Supplementary Data’ section.

### 2.2. Demographic Features of the Patients and Lesions Treated

In total, 224 patients across the eight papers were treated, of which 121 patients were treated with an Actifit scaffold and 103 had a CMI scaffold. Among these participants, 150 patients were male and 56 patients were female, but one of the papers, Schuttler et al. (2015), did not record the gender of the patients involved [28]. The lesions treated consisted of 173 medial (77%), 50 lateral (22%) and one bilateral meniscal injury. The mean patient ages ranged from 30.0 to 39.0 years. The minimum follow-up time was 24 months [24,25,26,28,29]. These findings are summarised in Table 1.

### 2.3. Clinical Outcome Measures and MRI Outcome Measures

Most of the studies included in this review used validated outcome measures which assess pain and function, for example the Visual Analog Scale (VAS), Knee injury and Osteoarthritis Outcome Score (KOOS), Lysholm score and Tegner activity level scale. However, there was a range of different scales that each study used.

In order to assess MRI findings, three scores were selected as inclusion criteria as mentioned in the ‘Methods’ section. Three studies used the International Cartilage Repair Society (ICRS) score [26,27,28] whilst five studies [22,23,24,25,29] used the Yulish score [31] in order to assess the effect of the implants on articular cartilage. Of the five that reported using the Yulish score, one study used a modified version [24]. All eight studies used the Genovese grading system [32] to assess the implanted scaffold itself [22,23,24,25,26,27,28,29]. However, unfortunately, of these eight studies, only six studies reported either exact grades for patients or average grades for both of the components of the Genovese score: Morphology and signal intensity compared to the normal native meniscus [23,24,25,26,27,29]. This is a limitation of the current literature, with the absence of full reporting of Genovese scores.

### 2.4. Second-Look Arthroscopy and Histological Evaluation of Biopsies

Only three of the eight studies used second-look arthroscopy to visualise the implant in situ and all of these studies were conducted by the same group [22,25,29]. One used Actifit, one used CMI and the other compared the two. In Bulgheroni et al. (2010), second look arthroscopy was only used in eight out of thirty-four patients and was only pre-planned in one. Four cases took place at 7M, one at 12M, one at 18M, one at 36M and one at 80M [22]. The procedure was performed in all other cases, due to possible complications. Bulgheroni et al. (2016) performed second look arthroscopy, at a range of 4-45M after surgery, in seven out of twenty-eight CMI and eleven out of twenty-five Actifit patients [29]. Bulgheroni et al. (2013) used second look arthroscopy in nine out of nineteen patients at both 12M and 18M [25]. All studies commented that, at the last second-look arthroscopy, the majority of patients showed a scaffold that was well integrated with the surrounding tissue, but was reduced in size in relation to the original scaffold [22,25,29]. Two studies also commented that the scaffold had irregular margins in relation to the original scaffold [25,29].

As with second look arthroscopy, only the same three papers performed a histological evaluation of the biopsies [22,25,29]. In Bulgerhoni et al. (2010), the biopsies used to evaluate the histology of the CMI scaffolds were taken during second look arthroscopy [22]. According to the authors, these specimens showed the development of fibrocartilaginous tissue with signs of increasing maturation as the time of follow-up increased. However, even five years after implantation, regeneration was not complete and differed from that of a normal meniscus. The regenerated tissue contained more cells in comparison to the natural meniscal tissue and also had more blood vessels whilst native meniscus is usually avascular. Bulgerhoni et al. (2013) used polarised light microscopy to show that at four months after the operation, fibroblastic and fibrochondrocytic cells had developed within the Actifit scaffolds, which further matured and became more organised with time [25]. In contrast to Bulgheroni et al. (2010), Bulgerhoni et al. (2013) found no evidence of vascularisation [22,25]. Bulgerhoni et al. (2016) confirmed the findings of both Bulgheroni et al. (2010) and Bulgheroni et al. (2013), stating that the CMI scaffolds had more fibrous tissue with fibroblastic cells and the development of blood vessels, whilst the Actifit biopsy samples showed no signs of vasculogenesis and the cells tended to be of a cartilaginous lineage, rather than fibroblastic [22,25,29]. All the studies found deposition of new tissue, initially in a heterogenous manner, with no signs of necrosis.

### 2.5. Complications and Treatment Failures

Seven of the eight studies reported a failure rate which ranged from 0% [28] to 31.8% [26]. The authors of this study with 31.8% failure rate, who used the Actifit scaffold, highlighted that the rate was higher for patients with a lateral compartment defect and suggested that the reason behind this might be because scaffolds are harder to apply in the lateral meniscus, as well as the fact that the lateral meniscus harbours a greater share of the load in its respective compartment compared to the medial meniscus. Six of the eight studies reported complications [22,23,24,25,27,29]. Swelling was the most common complication which affected a total of nine patients [22,23,24,29]. Only one of the eight studies, which used Actifit scaffolds, reported that there were no complications nor treatment failures [28]. Of the four studies that used Actifit and reported if they had complications, three had complications [24,25,29]. Of the four studies that used CMI and reported if they had complications, all four reported complications [22,23,24,29].

### 2.6. Do the Scaffolds Improve Clinical Outcomes?

Clinical outcome results were highly promising for both the Actifit and CMI studies. All showed statistical significance, in the very least, at one time point compared to baseline and one outcome measure from Lysholm, Tegner, VAS, KOOS subscores, IKDC objective and IKDC subjective, EQ-5D, UCLA, Kujala score, KSS function and KSS knee score.

Of the five studies that used Actifit, three studies reported that all clinical outcomes were statistically improved after their final follow-up period [26,28,29]. Of the three studies that reported outcomes at multiple post-operative time points, both Dhollander et al. (2016) and Bulgheroni et al. (2016) very encouragingly showed that at all time points improvements were statistically significant [26,29]. In contrast, Leroy et al. (2017) reported that after a 60M follow-up, only VAS and IKDC subjective scores were significantly improved for the intention to treat group (not for IKDC objective and KOOS subscores) and only the VAS, IKDC subjective, KOOS pain, KOOS daily activities and KOOS quality of life (not for IKDC objective, KOOS symptoms/stiffness and KOOS sport/recreation) for the per protocol group [27]. Bulgheroni et al. (2013) were able to show that clinical outcome improvements reached statistical significance at 6M and then were maintained over time [25].

The clinical outcomes for the CMI group were also highly encouraging. All four studies showed statistically significant improvements in all clinical outcome measures used when comparing final follow-up to baseline [23,24,25,29]. Of note, Zaffagnini et al. (2011), displayed that the statistical significance of clinical outcome score improvements was maintained over the 10-year period when compared to baseline, suggesting the longevity of the effects of the CMI implants in this case [23]. It also showed that compared to the control partial meniscectomy group, there were significant improvements in Tegner, VAS and IKDC objective, but not for the Lysholm score. The authors of this study suggested that the Lysholm score may not be sensitive enough as an outcome measure for the nature of this study. It should be stated, however, that this study also had a relatively small sample size, with seventeen patients staying for the full follow-up period. Bulgheroni et al. (2016) designed their study to compare patients receiving CMI to those receiving Actifit. It was highlighted by this study that, in terms of clinical outcome, there were no differences between the two groups after 24M, therefore suggesting that both Actifit and CMI have comparable effects on clinical outcome in patients [29].

### 2.7. Do the Scaffolds Have a Chondroprotective Effect?

The results regarding the chondroprotective effect of the scaffolds are conflicting and many of the papers do not have a long enough follow up period to accurately comment. Schuttler et al. (2011) and Leroy et al. (2017), using the Actifit scaffold, observed no significant changes in ICRS score and therefore showed no progression of cartilage damage, suggesting that although the scaffolds cannot reverse or improve cartilage status, they can inhibit the progression of disease [27,28]. In contrast, Dhollander et al. (2016) observed stable cartilage status at 5-year follow-up in only 46.7% of patients, which conflicts with the other authors’ observations and suggests that there is no chondroprotective effect of polyurethane Actifit scaffolds [26]. For the five papers which commented on cartilage integrity using the Yulish score, they all had similar findings showing either no significant changes or slight improvements throughout follow-up, which may suggest a chondroprotective effect [22,23,24,25,29]. In particular, Zaffagnini et al. (2011) showed the articular cartilage seemed to be preserved with the CMI compared to controls, however this did not reach significance [23]. Nevertheless, further studies with longer follow-up intervals are needed as all the papers commented on the unreliability of their results, mostly due to the short surveillance time.

### 2.8. How Are the Properties of the Scaffold Reflected in the Genovese Signal Intensity and Morphology Scores?

The ability of the scaffold to be resorbed was assessed using the Genovese morphology score, whilst the signal intensity of the scaffold on MRI may indicate both its porosity [29] and the maturation of the scaffold, as discussed further in the ‘Discussion’ section. 

Of the five studies that used the Actifit scaffold, two showed that after at least 24M, in the majority of patients in each study, the scaffold had a Grade 2 intensity: It was slightly hyperintense relative to the normal meniscus [25,29]. Leroy et al. (2017) also showed that after even 14.4M, the average scaffold intensity was 2 and this was maintained to 60M [27]. Therefore, according to these studies, the scaffold seems to have a tendency to remain slightly hyperintense throughout the follow-up and never reach the signal intensity of the native meniscus.

In comparison, there was only one study, Dhollander et al. (2016), that reported that the majority (60%) of patients had a Grade 1 scaffold, in other words, one that was markedly hyperintense compared to the native normal meniscus after 24M. The remaining 40% had Grade 2 scaffolds. This study went on to show that these results were maintained at 60M [26]. Due to this maintenance to 60M, both Dhollander et al. (2016) and Leroy et al. (2017) seem to agree that the Actifit seems to stabilise in signal intensity to this time point [26,27]. Unfortunately, Schuttler et al. (2011) did not report the percentages of patients who had Grade 1 or 2 signal intensity, but stated that all scaffolds achieved either a grade 1 or 2 signal intensity [28]. 

In relation to the morphology of the Actifit scaffold, two of the five studies showed that after at least 24M, in the majority of patients in each study, the scaffold had a Grade 2 morphology: It appeared to have irregular/regular morphology compared to the normal meniscus [26,29]. The remaining patients in these studies had Grade 3 scaffolds. Again, Leroy et al. (2017) showed that the average morphology grade was 2 after both 14.4M and 60M [27]. These studies therefore suggest that the Actifit scaffold tends to form irregular margins and reduce in size over time. In contrast, although Schuttler et al. (2015) did not report the numbers of patients for each grade, they instead reported that one patient had a grade 1 scaffold morphology after 24M [28]. This represented a total reabsorption of the scaffold that occurred between the 12M and 24M MRI images. Interestingly, this same patient reported a worsening of clinical outcome scores between this period and was the sole treatment failure in this study, due to their dissatisfaction with the procedure at 24M [28]. 

Genovese signal intensity results were varied for CMI as well. Of the four studies that used CMI implants, two reported that in the majority of the patients, a grade 1 markedly hyperintense signal was seen after 24M [22,24]. Interestingly, one of these two studies, reported that after another MRI at 60M, the majority of the patients now had grade 2 signal intensity scaffolds [22]. Bulgheroni et al. (2016) displayed that in the CMI group, all patients had a signal intensity of grade 2 after 24M, somewhat in accordance with the results of Zaffagnini et al. (2011) who showed that after both 60M and 120M, the median intensity score was 2 [23,29].

In terms of the morphology of the CMI implant, three studies showed that the majority of patients had Grade 2 implants after 24M [22,24,29]. Of note, Bulgheroni et al. (2016) were also able to show that an even greater majority of patients displayed a grade 2 implant after 60M [22]. Moreover, Zaffagnini et al. (2011) showed that the median morphology grade of implant was 2 after 60M, and these results were maintained at 120M [23]. Again, these studies together may suggest that the implant tends to develop irregular margins and reduce in size with time, but this was not proven in these studies.

## 3. Discussion

### 3.1. Quality of Included Studies

In this review, eight studies published between 2010 and 2017 were included. The mean CMS suggests that on the whole, the quality of the studies was good. The average CMS score for Part A was only 52% of the maximum compared to Part B which was 78%. This suggests that the studies included were better at defining their outcome criteria properly and assessing their outcome criteria in an unbiased manner than improving parameters of the study design, such as the number of patients included, follow-up period and type of study. Indeed, an inherent limitation with this review and where the studies tended to score poorly in, was the fact that six out of eight studies were case series with no control groups and five out of eight studies had a sample size of fewer than 30 patients. Moreover, only two studies had a follow-up period of more than 61 months and thus this review focuses on the effect of scaffolds on the outcome in the short to mid-term. Further studies must be conducted with control groups, longer follow-up periods and more patients.

### 3.2. How do the Scaffolds Perform in Relation to Clinical Outcomes?

Due to the variability in results and the quality of studies, it is difficult to determine the success of meniscal scaffolds. Both the lowest and highest failure rates were with Actifit scaffolds [26,28] and the definition of failure was not standardised between studies, hence they are difficult to use as a measure of success. Clinical outcomes were promising, with statistical improvements seen in at least one outcome measure, comparing at least one time point to baseline. However, there were measures that did not show significant improvement, including the Lysholm score when Zaffagnini et al. (2011) compared CMI to the control partial meniscectomy [23]. Leroy et al. (2017) reported that only certain measures significantly improved at 60M follow up with Actifit [27]. Nevertheless, in both papers the majority of outcome measures did show improvement and explanations have been suggested by the authors, such as the Lysholm score not being sensitive enough [23]. Overall, the clinical outcomes were positive and showed the benefits of both CMI and Actifit scaffolds.

### 3.3. Is Initial Porosity and Maturation of the Scaffolds Evident after Implantation?

Scaffolds have been designed to have an even distribution of sizeable pores that are interconnected [18]. This allows for tissue infiltration and for cell media and growth factors to travel through the scaffold. However, a high porosity may also lead to weaker mechanical properties; some evidence suggests that less porous scaffolds produce mechanical properties that are more similar to the native meniscus, as seen in compression tests, as well as stress relaxation tests [33]. Therefore, the pore size must be optimised for the specific tissue involved [19]. The Genovese grading system assessed the signal intensity on MRI. For both the Actifit and CMI scaffolds, the majority of studies reported that the majority of patients had scaffolds of Grade 1 or 2 intensity or an average/median grade of 2, showing that the scaffold was either slightly or, less frequently, markedly hyperintense compared to the native normal meniscus [25,26,27,29]. This could be due to resorption of the scaffold, but as Bulgheroni et al. [29] suggests that this could also be due to the porous nature of scaffolds, which was also suggested by De Coninck et al. [34]. The slight diminishing of intensity with time is expected with tissue ingrowth and collagen production, but the intensity does not decrease to the level of the native meniscus. Others have also suggested that the hyperintense signal may be related to maturation of the scaffold [35]

### 3.4. Do Scaffolds Resorb Well, as Assessed by MRI?

Scaffolds have been created to be resorbed upon the integration of meniscal tissue. Giant cells resembling osteoclasts engulf collagen fibres from the CMI and it is largely resorbed by 12-17 months in canines [14]. Macrophages engulf the polyurethane segments in Actifit, whilst the polycaprolactone has ester bonds that are hydrolysed and determine the degradation rate of the scaffold. Polyurethane has been shown to be resorbed both in vitro and in vivo [16,36]. Morphology of the scaffold, as assessed by MRI, was also looked at by the Genovese grading system. Grade 1 on the Genovese scale indicates complete resorption of the scaffold [32]. Although one patient was reported as having grade 1 scaffold morphology [28] and Bulgheroni et al. (2013) found that the average morphology grade was 1.3 after 12M [25], two Actifit studies showed that in the majority of patients, a grade 2 morphology was seen after 24M [26,29] and in another, an average grade 2 morphology was found after 60M [27]. Similarly, most studies for the CMI implant showed that a majority of patients had a grade 2 morphology [22,24,29]. Both the CMI and Actifit scaffolds have been developed to be resorbed or biodegraded over time, so some resorption is expected and the tendency to form irregular margins and reduce in size is a demonstration of this. However, it is essential that enough tissue ingrowth has occurred by this point so the longer a scaffold maintains its size and morphology, the better the results should be. It is important to acknowledge that the use of the Genovese scale for MRI assessment of collagen meniscus implants has been called into question, due to low inter and intra-observer reliabilities [37]. Therefore, caution should be taken when making any conclusions based on these scores.

### 3.5. Do the Scaffolds Have Chondroprotective Properties?

All five of the studies that used the Yulish score to assess the articular cartilage after implant showed no significant changes or slight improvements throughout follow-up, which may suggest a chondroprotective effect of the scaffold [22,23,24,25,29]. However, this interpretation is clouded by the limited follow-up used in these studies. Further, as detailed in the results section, the studies which used the ICRS score produced conflicting results as to the assessment of any cartilage damage with the Actifit scaffold [26,27,28]. 

It is important to consider that the Yulish score was originally designed for assessment of the posterior patellar hyaline cartilage in chondromalacia patellae [31] and the ICRS was not used in any CMI studies. Along with small sample sizes and short-term follow-up, this means it is difficult to determine a chondroprotective effect via MRI. In addition, post-operative MRI findings are yet to be found to correlate with either second-look arthroscopy findings or histological appearance of the scaffold in humans, as commented on by Zaffagnini et al. [35]. In all, although MRI does provide a non-invasive method of evaluating the scaffold, which is more practical than second-look arthroscopy, it is important to establish whether there is enough correlation in humans and clinical outcomes to justify its use in these studies. Recent years have seen the development of quantitative MRI techniques and studies have shown direct correlations between T1rho, T2 values and the biochemical composition/histology of human cartilage in osteoarthritis [38]. Whether this can be applied to correlate histology and MRI values and therefore evaluate articular cartilage after meniscus implant could be explored in the future.

### 3.6. Are the Regenerative Properties of the Scaffolds Evident?

Previous reviews have detailed how the scaffold should be designed to allow for the regeneration of the tissue [19]. Tissue regeneration can be encouraged by the mechanical stimuli scaffolds produce. Biologically, they should be porous enough such that cell media and growth factors can reach cells throughout the scaffold. They should also act as a vehicle for growth factor delivery. 

The blood vessel infiltration of the Actifit scaffold allows for tissue ingrowth. This tissue ingrowth has been previously demonstrated in vivo, with the presence of fibro-vascular tissue [18]. In the included studies, however, Bulgerhoni et al. found no signs of vasculogenesis [25,29]. Despite this, Bulgerhoni et al. (2013) found that fibroblastic and fibrochondrocytic cells had developed within the Actifit scaffolds, which further matured and became more organised with time [25]. These results are in contrast to other studies, which noted the presence of vascularised tissue layers, containing mainly fibroblasts and fusiform fibrochondroblast-like cells [39]. 

The CMI scaffolds are made from type I collagen with glycosaminoglycans and its fibres are swollen in the presence of equal parts hyaluronic acid and chondroitin sulphate [40]. The hyaluronic acid has previously been indicated to suppress nitric oxide production in animals and nitric oxide donors have been shown to cause meniscal degeneration. Thus, it follows that HA helps meniscal healing [11,12]. The presence of glycosaminoglycans also helps with cellular growth [41]. Histologically, Bulgheroni et al. (2016) noted that CMI scaffolds had more fibrous tissue and fibroblastic cells with the development of blood vessels [29]. Bulgheroni et al. (2010) showed that the biopsies demonstrated the development of fibrocartilaginous tissue resembling meniscus, including meniscal cell types [22]. This also matured over time. This similarity has been seen on a biochemical level previously too; Stone et al. looked at collagen scaffolds in an animal study and studied chromatographic profiles [13]. They found that the proteoglycans produced by the regenerated menisci were phenotypically similar to ones made by the natural meniscus. Together with other results, they suggest that this represented the formation of normal, healthy, fibrocartilginous menisci. 

Having said this, it is important to note that only three of the included eight studies conducted a histological evaluation [22,25,29]. Therefore, any conclusions from this data must be put into perspective. Thus, although initial results may indeed be promising, without further exploration, it is difficult to ascertain any real conclusion on regeneration. 

### 3.7. Are the Integrative Properties of the Scaffolds Evident?

Scaffolds are also designed to have the capacity for host-tissue integration [19]. Integration with the surrounding tissue is an important aspect of any scaffold. For example, the Actifit polymer consists of polycaprolactone and urethane segments. The urethane segments are thought to be more stable than the polycarprolactone segments and so have to be integrated into the surrounding tissue or phagocytozed by giant cells and macrophages [16,36,42].

To assess the integration of the scaffolds, studies used second look arthroscopy. The included studies showed that the majority of patients had a scaffold that was well integrated with the surrounding tissue. It is again however unfortunate that only three studies conducted a second-look arthroscopy [22,25,29]. This method is the gold standard when assessing the integration of the scaffold and therefore more studies in the future should aim to utilise such a method wherever possible and practical [22].

### 3.8. Limitations

This study had several limitations. Only four databases were searched (PubMed, EMBASE, Scopus and clinicaltrials.gov) and only studies in English were screened. It is essential to highlight that the studies in this review were mostly of low-level evidence, such as case series and that there was significant variability in study methodology between studies thus preventing the direct comparison of the studies included. The systematic review included only eight studies and therefore represents a small subset of the literature on meniscal scaffolds. Further, of the eight studies, five studies originated from two of the same centres, thus introducing possible bias into the sample [22,23,24,25,29]. Additionally, this review included studies with concomitant procedures, which could in part, have contributed to their results along with the meniscal scaffolds [43].

### 3.9. The Future of Meniscal Scaffolds

It is clear from the discussion above that a big challenge for this field is to agree on which clinical or radiological outcomes are best suited to assessing the efficacy of these scaffolds. Without this standardisation, sample sizes per outcome measure are too small to allow for powerful comparison and evaluation. A primary aim of scaffold implantation is to normalise mechanical loading and prevent articular cartilage degeneration [22]. Therefore, we call for more studies to investigate not only the integrity of the scaffold, but also the effect of the scaffold on the articular cartilage, as few papers assessed both. Along with more standardised research on the two currently available scaffolds, it is important that we look to other potential scaffold compositions. Tissue derived materials typically lead to more successful tissue regeneration, but there are issues with creating the correct pore sizes and having adequate mechanical strength [44]. Polycarbonate-urethane has been looked into as it is a strong polymer with relatively high elasticity allowing both mechanical strength and conformity. It has shown positive results in vitro and is now being used in humans in certain countries, thus far producing mixed results [45,46,47]. Biopolymers are a potential alternative to polymers, such as polyurethane and polycaprolactone which are used in Actifit. One example is silk fibroin from silk worms, with seemingly improved biocompatibility and mechanical properties in comparison to non-biological polymers and increased similarity to the native meniscus [48,49,50]. A composite 3:1 ratio of silk fibroin and polyvinyl alcohol was shown to have good tissue ingrowth and ECM secretion in vitro, as well as being nontoxic in vivo [51]. Hydrogels also have important potential and have been shown to promote higher levels of ECM secretion than polycaprolactone alone. GelMA promoted collagen synthesis and agarose/MeHA promoted GAG synthesis, the former important for cell differentiation of the inner meniscus and the latter important for the outer [52]. The key point to take from this is that there are many potential materials that could generate a successful scaffold, as well as a multitude of growth factors and additives that could support tissue growth and delay degradation. We hope to see these avenues explored in more detail and replicated so that innovative tissue engineering can be quickly advanced to a clinical setting.

## 4. Methods

For this study, a literature search using PubMed, EMBASE, Scopus and clinicaltrials.gov was performed in November 2018. The search terms included ‘menisc*’ AND ‘scaffold OR implant OR replacement OR engineering’ AND ‘knee’. The inclusion criteria were: human clinical results; published in a peer-reviewed journal; using a meniscal scaffold to repair a medial or lateral meniscal tear; assessing the scaffold treatment using clinical outcomes; assessing scaffold treatment with MRI using Genovese score [32] with either Yulish [31] or ICRS score [53]. Studies had to clearly describe their results for the Genovese scale and for either the Yulish score or ICRS score. Exclusion criteria included all allograft studies, preclinical studies (including in vitro and animal studies), case studies, studies with fewer than ten patients and studies for which the full text is not freely available or not published in the English language.

Initial screening of the papers consisted of reviewing the titles found by the initial search. The abstracts of the papers deemed relevant in the initial screen were found and assessed by four authors (DS, JCR, CDSR and CKIR) to see if they met the inclusion criteria. A hand search was performed of relevant review articles to identify any further articles which met the criteria. The full-texts of the papers selected were then analysed.

To assess the quality of the studies, the Coleman Methodology Score (CMS) was utilised [30]. Each study was checked against the criteria of the CMS and scores were subsequently calculated. The review was written in accordance with the PRISMA guidelines [21].

## 5. Conclusions

This systematic review shows that studies should report the evaluation of both the scaffold and its effect on articular cartilage more clearly, whilst also highlighting the lack of higher-level evidence and the need for standardisation of outcome measures across the field with longer follow-up times. Further, studies should assess both clinical and MRI outcomes, as well as use histological assessment. Only once this is achieved can we obtain a realistic sense of whether the scaffolds achieve any of the ideal biological and biomechanical properties in clinical practice. Initial results, however, indicate that the scaffolds do produce positive clinical outcomes and some authors suggest that they integrate well into the surrounding tissues. Some studies have also suggested that they maintain porosity after implantation, resorb successfully, mature and encourage regeneration of the tissue with chondroprotective effects. Indeed, newer techniques, such as quantitative MRI may help to evaluate this better in the future and assess newer scaffolds that are currently being designed with more refined properties, when they eventually arrive at the door of randomised controlled trials. 

## Figures and Tables

**Table 1:** The study demographics and details.

Author	Year	Type of Meniscal Implant or Scaffold	Number of Patients	Mean Patient Age (Y)	Male:Female	Type of Lesion: Medial or Lateral	Mean Follow-up (Range) (M)	Details of Concomitant Procedure
Bulgheroni [25]	2013	Actifit	19	32.8	17:2	16 medial, 2 lateral, 1 bilateral	24 (24–46)	17 patients: 8 ACL reconstructions, 1 ACL repair, 7 high tibial osteotomies and 1 femoral osteotomy
Dhollander [26]	2016	Actifit	44	32.1	24:20	29 medial and 15 lateral	60 (24–60)	8 patients: 4 ACL reconstructions and 4 high tibial osteotomies
Leroy [27]	2017	Actifit	15	30.0	8:7	6 medial 9 lateral	72 (60–97.2)	6 patients: 5 ACL reconstructions, 1 mosaicplasty
Schuttler [28]	2015	Actifit	18	32.5	NR	18 medial	NR - minimum 24	No patients
Bulgheroni [22]	2010	CMI	34	39.0	25:9	34 medial	NR (60–76)	14 patients: 11 ACL reconstructions, 2 high tibial osteotomies and 1 microfracture
Zaffagnini [23]	2011	CMI	17	38.0	17:0	17 medial	135 (120–152)	2 patients: 2 ACL reconstructions
Zaffagnini [24]	2012	CMI	24	36.3	20:4	24 lateral	26 (24–31)	11 patients: 4 ACL reconstructions, 6 lateral tibial plateau microfractures, 1 ACL cyst removal
Bulgheroni [29]	2016	CMI and Actifit	28 CMI; 25 Actifit	38.7 CMI; 34.4 Actifit	19:9 CMI; 20:5 Actifit	53 medial	NR—minimum 24	Actifit—25 patients: 11 tibial osteotomies, 9 ACL reconstructions, 3 microfractures, 1 healing response, 1 suture CMI—15 patients: 3 tibial osteotomies, 11 ACL reconstructions and 1 microfracture

ACL: Anterior Cruciate Ligament, CMI: Collagen Meniscus Implant, M: months, NR: not reported, Y: years.

**Table 2:** The clinical outcome results of each study.

Author	Year	Clinical Scores (Mean ± Standard Deviation)					OR Description of Main Clinical Results
		0M	6M	12M	24M	60M	
**ACTIFIT**							
Bulgheroni [25]	2013	Lysholm: 66.2	86.8*	90.7	90.5	-	Clinical outcome scores were significantly improved after 6M compared to baseline.
		Tegner: 3.8	4.1*	4.9	6	-	
		VAS: 6.23	3.07*	2.1	1.94	-	
Dhollander [26]	2016	KOOS Total: 206.5 ± 79.7	-	-	329.8 ± 108.9*	333.6 ± 112.2*	All post-operative clinical outcome scores showed statistically significant improvement when compared to baseline.
		KOOS Pain: 48.3 ± 20.3	-	-	72.9 ± 23.6*	77.2 ± 24.5*	
		KOOS Symp/stiffness: 52.4 ± 19.7	-	-	73.2 ± 18.4*	69.4 ± 20.9*	
		KOOS ADL: 54.4 ± 21.5	-	-	77.1 ± 23.9*	80.2 ± 26.1*	
		KOOS sport/rec: 19.1 ± 20.0	-	-	57.0 ± 35.6*	49.7 ± 34.8*	
		KOOS QOL: 32.2 ± 14.2	-	-	49.6 ± 21.6*	56.9 ± 24.0*	
		VAS for pain: 56.2 ± 21.6	-	-	24.6 ± 22.7*	19.3 ± 26.9*	
		IKDC subjective form: 38.7 ± 14.8	-	-	63.4 ± 24.3*	66.9 ± 23.1*	
Leroy [27]	2017	Intention to treat group					
		VAS: 5.5 ± 2.0	-	3.7 ± 2	2.9 ± 2.1	2.9 ± 2.6*	Intention to treat group: VAS and IKDC subjective score statistically improved from baseline to final post-operative timepoint, with no other measures showing statistical improvement.
		IKDC subjective: 51.2 ± 20	-	62.1 ± 19	65.1 ± 22	66.1 ± 23	
		IKDC objective: 2A/6B/3C/2D	-	4A/5B/3C/1D	3A/2B/7C/1D	2A/7B/3C/1D	
		KOOS Symp/stiffness: 69.4 ± 13	-	75 ± 14	76.5 ± 15	68.3 ± 23	
		KOOS pain: 62.9 ± 15	-	75 ± 16	77.8 ± 18	76.1 ± 25	
		KOOS ADL: 72 ± 20	-	79.7 ± 14	83.5 ± 17	81.7 ± 23	
		KOOS Sport/rec: 51.2 ± 14	-	55.8 ± 21	61.2 ± 17	53.5 ± 33	
		KOOS QOL: 40.9 ± 18	-	57.7 ± 27	64.3 ± 25	59.9 ± 31	
		Per protocol group					
		VAS: 5.3 ± 2.2	-	-	-	1.9 ± 1.7*	Per protocol group: VAS, IKDC subjective, KOOS pain, KOOS daily activities and KOOS QOL were statistically improved from baseline to final post-operative time point with no other measures showing statistical improvement
		IKDC subjective: 49.6 ± 19	-	-	-	75.4 ± 15*	
		IKDC objective: 1A/5B/2C/2D	-	-	-	2A/6B/2C/0D	
		KOOS symp/stiffness: 67.1 ± 14	-	-	-	76.3 ± 16	
		KOOS pain: 60.6 ± 16	-	-	-	86 ± 13*	
		KOOS ADL: 70.3 ± 21	-	-	-	90.2 ± 11*	
		KOOS sport/rec: 50.5 ± 14	-	-	-	65.5 ± 25	
		KOOS QOL: 42.7 ± 17	-	-	-	71.1 ± 25*	
Schuttler [28]	2015	KOOS pain: 47 ± 14.5	KOOS pain: 75 ± 17.7*	KOOS pain: 82 ± 17.4*	KOOS pain: 83 ± 18.6*	-	All KOOS subdomains showed statistically significant improvements comparing preoperative and 24M scores. KSS function and knee both showed statistically significant improvement over whole time period. VAS: Statistically significant reduction compared to baseline at every time point. UCLA: Statistically significant increase compared to baseline was only seen at 2 years.
		KOOS symp/stiffness: 60 ± 16.2	KOOS symp/stiffness: 67 ± 18.5*	KOOS symp/stiffness: 85 ± 9.7*	KOOS symp/stiffness: 81 ± 13.4*		
		KOOS ADL: 53.1 ± 16.0	KOOS ADL: 85 ± 14.5*	KOOS ADL: 88 ± 13.0*	KOOS ADL: 91 ± 14.7*		
		KOOS Sport/rec:26 ± 20.5	KOOS Sport/Rec: 60 ± 25.3*	KOOS Sport/Rec: 68 ± 24.5*	KOOS Sport/Rec: 66 ± 28.5*		
		KOOS QOL: 28 ± 16.6	KOOS QOL: 55 ± 26.9*	KOOS QOL: 67 ± 20.4*	KOOS QOL: 63 ± 18.9*		
		KSS function score: 61 ± 22.2	KSS function score: 87 ± 10.2*	KSS function score: 89 ± 15.7*	KSS function score: 96 ± 7.9*		
		KSS knee score: 65 ± 9.4	KSS knee score: 89 ± 13.1*	KSS knee score: 87 ± 14.1*	KSS knee score: 88 ± 12.4*		
		UCLA: 5.4 ± 1.8	UCLA: 6.1 ± 1.8	UCLA: 6.5 ± 2.1	UCLA: 7.3 ± 1.8*		
		VAS: 5.1 ± 2.0	VAS: 2.1 ± 2.4*	VAS: 1.8 ± 2.3*	VAS: 1.5 ± 2.1*		
**CMI**							
Bulgheroni [22]	2010	Lysholm: 58	-	-	94*	-	Both clinical outcome scores showed statistically significant improvements comparing 24M to baseline.
		Tegner: 2	-	-	5*	-	
Zaffagnini [23]	2011	-	-	-	-	-	Comparing 10-year follow-up to baseline, statistically significant improvements seen in all clinical outcome measures: VAS, Lysholm, SF-36 PHI, SF-36 MI, IKDC and Tegner scales.
Zaffagnini [24]	2012	Lysholm: 64 ± 16.2	89.9 ± 11.4*	-	92.7 ± 13.8*	-	All clinical outcome scores showed statistically significant improvements from baseline to last post-operative follow up.
		VAS: 55.2 ± 29.4	18.3 ± 18.1*	-	19.5 ± 25.6*	-	
		Tegner: 3 (median)	4 (median)	-	5 (median)*	-	
		Objective IKDC: 6A, 14B, 4C, 0D	20A, 3B, 0C, 1D*	-	20A, 3B, 0C, 1D*	-	
		EQ-5D: 0.579 ± 0.28	-	-	0.892 ± 0.14*	-	
**ACTIFIT VS CMI**							
Bulgheroni [29]	2016	CMI Group					
		Lysholm: 58.4 ± 17.3	-	92.5 ± 8.5*	94.5 ± 6.0* (at minimum 24M follow-up)	-	In both CMI and Actifit groups, all clinical outcome scores showed a statistically significant improvement across the follow-up period when compared to baseline. However, none of the differences between values at 12M and final-follow up reached statistical significance.
		Tegner:2	-	5*	5* (at minimum 24M follow-up)	-	
		Actifit Group					
		Lysholm: 67.0 ± 15.7	-	87.4 ± 13.0*	90.3 ± 13.1* (at minimum 24M follow-up)	-	As above
		Tegner:4	-	4*	5* (at minimum 24M follow-up)	-	

All scores reported as mean ± standard deviation unless otherwise stated. * = significant difference to baseline (*p* < 0.05). CMI: Collagen Meniscus Implant, EQ-5D: EuroQol 5 dimensions, IKDC: International Knee Documentation Committee Score, KOOS ADL: Knee injury and Osteoarthritis Outcome Function in daily life score, KOOS QOL: Knee injury and Osteoarthritis Outcome Quality of Life score, KOOS Sport/rec: Knee injury and Osteoarthritis Outcome Function in Sport and Recreation score, KOOS Symp/stiffness: Knee injury and Osteoarthritis Outcome Symptom score, KSS: Knee Society Score, M: months, UCLA: University of California, Los Angeles activity scale, VAS: Visual Analog Scale.

**Table 3:** The MRI outcome results of each study.

Author	Year	MRI Findings									
		Yulish Score at Follow-up (M)				ICRS Grade at Follow-up (M)				Genoverse Grading	
		0	24-120M	Final Follow-up	Or Description of Yulish Results	0	24	60	Or Description of ICRS Grade Finding	Genovese Morphology at Follow-up	Genovese Signal Intensity at Follow-up
**ACTIFIT**											
Bulgheroni [22]	2013	1	At 24M: 1.1		Articular cartilage was not degenerated					12 M: 1.3 24 M: 1.4 Scaffolds did not change in morphology.	12M: Grade 2 24 M: Grade 2
Dhollander [26]	2016					Patient 1: 2	Patient 1: 2	Patient 1: 3a	Cartilage of the index compartment remained stable in 46.7% of patients after 5 years. 1 patient showed a temporary improvement in cartilage after 2 years which then deteriorated at 5-year follow-up. The remaining 46.7% patients showed a deterioration in cartilage. A chondroprotective effect was therefore not seen.	24M and 60M: Grade type 2b in all patients	24M and 60M: Grade 1 in 60% of patients, grade 2 in 40% of patients
						Patient 2: 2	Patient 2: 1	Patient 2: 3a			
						Patient 3: 0	Patient 3: 1	Patient 3: 2			
						Patient 4: 1	Patient 4: 1	Patient 4: 2			
						Patient 5: 1	Patient 5: 1	Patient 5: 3a			
						Patient 6: 1	Patient 6: 1	Patient 6: 1			
						Patient 7: 0	Patient 7: 0	Patient 7: 1			
						Patient 8: 0	Patient 8: 0	Patient 8: 0			
						Patient 9: 0	Patient 9: 0	Patient 9: 0			
						Patient 10: 1	Patient 10: 2	Patient 10: 2			
						Patient 11: 0	Patient 11: 0	Patient 11: 3b			
						Patient 12: 0	Patient 12: 0	Patient 12: 0			
						Patient 13: 0	Patient 13: 0	Patient 13: 0			
						Patient 14: 0	Patient 14: 0	Patient 14: 0			
						Patient 15: 0	Patient 15: 0	Patient 15: 0			
Leroy [27]	2017					6 patients with Grade 0.			Mean ICRS grade was stable pre-operatively to 60M follow-up.	14.4M and 60M: Average Grade 2	14.4M and 60M: Average Grade 2
						1 patient with Grade 1.					
						3 patients with Grade 2.					
						3 patients with Grade 3.					
						2 patients with Grade 4.					
Schuttler [28]	2015								89% of patients showed stable articular cartilage from 6 months to 24 months follow-up. 11% of patients showed interval progression of 1 or more ICRS grades from 6 months to 24 months follow-up. 27.8% of patients showed improvements in chondral wear after 24 months. Overall, there was no significant difference in cartilage damage comparing consecutive MRIs.	1 patient showed Grade 1 after 24M. All other results not specified.	24M: All patients showed Grade 1 or Grade 2 signal intensity. (numbers not specified)
**CMI**											
Bulgheroni [22]	2010		At 24M: Grade 0 in 67% of patients, Grade 1 in 14% of patients, Grade 2 in 4% of patients, Grade 3 in 4% of patients, Grade 4 in 11% of patients.	At 60M, Grade 0 in 60% of patients, Grade 1 in 14% of patients, Grade 2 in 11% of patients, Grade 3 in 4% of patients, Grade 4 in 11% of patients.	No signs of degeneration of the articular cartilage of medial compartment.					2Y: Grade 2 in 60.7% of patients 5Y: Grade 2 in 71.4% of patients. The remaining patients had Grade 3 morphology.	2Y: Majority of patients showed Grade 1 intensity 5Y: Majority of patients showed Grade 2 intensity (percentages not specified)
Zaffagnini [23]	2011	2 ± 1.5 (median)	At 120M: 2 (median)		Articular cartilage seemed to be preserved across the follow-up period, however this did not reach statistical significance					At 60M and 120M: 2 (median)	At 60M and 120M: 2 (median).
Zaffagnini [24]	2012	Lateral Tibial Plateau: Modified score: 1.5 (1.0-2.0) (median, IQR) Lateral Femoral Condyle: Modified score: 2.0 (1.0-2.0) (median, IQR)		Lateral Tibial Plateau: Modified score: 1.0 (1.0-0.5) (median, IQR). Lateral Femoral Condyle: Modified score: 1.5 (1.0-2.0) (median, IQR)	Mean modified Yulish scores showed an improvement comparing baseline to final post-operative scores for both lateral femoral condyle and lateral tibial plateau, however this did not reach statistical significance					24M: 75% had Grade 2, 12.5% had Grade 1, 12.5% had Grade 3	24M: 50% of patients had Grade 2, 37.5% had Grade 3 (remaining not specified)
**ACTIFIT VS CMI**											
Bulgheroni [29]	2016	CMI patients: Preoperatively: 67.9% of patients showed Grade 0, 14.3% showed Grade 1, 3.6% showed Grade 2, 3.6% showed Grade 3, 10.7% showed Grade 4. Actifit patients: Preoperatively: 16% of patients showed Grade 0, 36% showed Grade 1, 32% showed Grade 2, 12% showed Grade 3, 4% showed Grade 4.	CMI patients: 2 years: 60.7% of patients showed Grade 0, 14.3% showed Grade 1, 10.7% showed Grade 2, 3.6% showed Grade 3, 10.7% showed Grade 4. Actifit patients: 2 years: 20% of patients showed Grade 0, 44% showed Grade 1, 20% showed Grade 2, 16% showed Grade 3.		Therefore, there was no statistically significant evidence of degeneration over the follow-up period.					CMI patients: Genovese morphology: 61% patients Grade 2, 39% patients Grade 3, 1 case Grade 1 (complete resorption of the scaffold). Actifit patients: Genovese morphology: 79% patients Grade 2, 21% patients Grade 3.	CMI patients: Genovese signal intensity: 54% patients Grade 2A, 46% patients Grade 2B Actifit patients: Genovese signal intensity: 68% patients Grade 2A, 38% patients Grade 2B (number as stated in paper).

ICRS: International Cartilage Repair Society score, MRI: Magnetic Resonance Imaging, M: Months, Y: Years.

**Table 4:** The second-look arthroscopy and histological findings along with complications and failure rate of each study.

Author	Year	Second Look Arthroscopy	Histology	Complications/Failure Rate
**ACTIFIT**				
Bulgheroni [25]	2013	9/19 patients had second look arthroscopy. Performed at 12M and 18M. 12M: scaffold was well integrated, reduced in size. 18M: scaffold was reduced in size compared to original scaffold, irregular margin.	4M: new heterogenous tissue formed made up of fibroblastic cells 36M: the tissue was more organised Vessels were never found	Complication: knee stiffness in 1 patient with ACL reconstruction + scaffold Failure rate: 1/19 (5.3%).
Dhollander [26]	2016	N/A	N/A	Failure rate: 14/44 (31.8%)
Leroy [27]	2017	N/A	N/A	Complications: 3 complications Failure rate: 3/15 (20%)
Schuttler [28]	2015	N/A		Complications: None. Failure rate: 0/18 (0%)
**CMI**				
Bulgheroni [22]	2010	8/34 patients had second look arthroscopy. Performed at either 7M (4 cases), 12M, 18M, 36M, 80M. All showed scaffold was well integrated, reduced in size compared to original scaffold.	Biopsies were taken for histological analysis during second look arthroscopy. 36M: meniscoid tissue with vasculogenesis 60M: fibres of the scaffold were completely absorbed. Two different connective tissues were observed - one more compact and the other looser	2 complications: 1. suture tied with infrapatellar branch of the saphenous nerve 2. continuous swelling in knee Failure rate: 2/34 (5.8%)
Zaffagnini [23]	2011	N/A	N/A	Complications: Swelling in 1 case and pain with swelling in 1 case. Failure rate: 2/17 (12%)
Zaffagnini [24]	2012	N/A	N/A	Complications: No complications reported that were related to the implant. 1 patient had arthroscopic debridement 6 months after implantation as they reported knee pain and swelling. Failure rate: 1/24 (4%)
**ACTIFIT VS CMI**				
Bulgheroni [29]	2016	7/28 CMI patients and 11/25 Actifit patients had second look arthroscopy. Performed at range of 4-45M. In all but 1, scaffold was well integrated, reduced in size compared to original scaffold, irregular margin. 1 CMI patient had complete resorption of scaffold.	CMI: fibrous tissue, vascular Actifit: fibrocartilaginous, avascular Both: developed over time, showed no signs of necrosis	CMI Group: 3 complications: neuro apraxia of infrapatellar branch of the saphenous nerve, persistent synovitis, superficial infection. Actifit Group: 5 complications: 1 case of joint stiffness, 4 cases of synovitis. Failure rate: not mentioned.

M: Months, N/A: Not applicable.

## Supplementary Materials

The following are available online at http://www.mdpi.com/1422-0067/20/3/632/s1.

## Abbreviations

ACL	Anterior Cruciate Ligament
CMI	Collagen Meniscus Implant
CMS	Coleman Methodology Score
*ECM*	Extracellular Matrix
*EQ-5D*	EuroQol 5 dimensions
*GelMA*	Gelatin methacrylamide
*GAG*	Glycosaminoglycan
*MeHA*	Methacrylated hyaluronic acid
HA	Hyaluronic Acid
ICRS	International Cartilage Repair Society
*IKDC*	International Knee Documentation Committee Score
*KOOS ADL*	Knee injury and Osteoarthritis Outcome Function in daily life score,
*KOOS QOL*	Knee injury and Osteoarthritis Outcome Quality of Life score
*KOOS Sport/rec*	Knee injury and Osteoarthritis Outcome Function in Sport and Recreation score
*KOOS Symp/stiffness*	Knee injury and Osteoarthritis Outcome Symptom score, *KSS* Knee Society Score,
*KSS*	Knee Society Score
M	Months
MAT	Meniscal Allograft Transplantation
MRI	Magnetic resonance imaging
*N/A*	Not Applicable
NO	Nitric Acid
NR	Not Reported
PRISMA	Preferred Reporting Items for Systematic Reviews and Meta-Analyses
*UCLA*	University of California, Los Angeles activity scale,
*VAS*	Visual Analog Scale
Y	Years
