# Supplementary material for: Are the Biological and Biomechanical Properties of Meniscal Scaffolds Reflected in Clinical Practice? A Systematic Review of the Literature
Source: Int J Mol Sci. 2019 Feb 1;20(3):632. doi: 10.3390/ijms20030632 (PMC6386938; doi:10.3390/ijms20030632)
Supplement: Supplementary file 1 [file ijms-20-00632-s001.pdf]

Table S1. The Coleman methodology scores for all eight studies.

Authors	Year	Part A (max = 65)						Part B (max = 35)										Total
		**1**	**2**	**3**	**4**	**5**	**6**	**1**				**2**				**3**		
Bulgheroni [26]	2010	4	7	10	0	10	5	2	2	3	0	5	0	3	3	5	5	64
Bulgheroni [29]	2013	0	4	10	0	10	5	2	2	0	0	5	0	3	3	5	5	54
Bulgheroni [33]	2016	7	4	7	10	10	5	2	2	0	0	5	0	3	3	5	5	68
Dhollander [30]	2016	4	7	10	0	10	5	2	2	0	0	5	4	3	3	5	5	65
Leroy [31]	2017	0	10	10	0	10	5	2	2	0	0	5	0	3	3	5	5	60
Schuttler [32]	2015	0	4	10	0	10	0	2	2	0	0	5	0	3	3	5	5	49
Zaffagnini [27]	2011	0	10	10	10	10	0	2	2	0	3	5	0	3	3	5	5	68
Zaffagnini [28]	2012	0	4	10	0	10	5	2	2	0	3	5	4	3	3	5	5	61
